# Comparative Effectiveness of Immersive Virtual Reality and Traditional Didactic Training on Radiation Safety in Medical Professionals: A Crossover Study

**DOI:** 10.1002/jmrs.867

**Published:** 2025-03-03

**Authors:** Wanjiku Mwangi, Yuki Tanaka

**Affiliations:** ^1^ Osaka University Hospital Suita Osaka Japan

## Abstract

**Introduction:**

Radiation safety is critical in medical settings where ionising radiation is routinely used. Traditional didactic training methods often fail to provide the practical skills needed for effective safety protocol implementation. This study aimed to compare the effectiveness of virtual reality (VR)‐based radiation safety training with traditional didactic methods in reducing radiation exposure among medical professionals. Secondary objectives included assessing participant satisfaction, engagement and confidence in applying radiation safety practices.

**Methods:**

A 2‐year randomised crossover trial was conducted with 39 medical professionals from cardiac catheterization laboratories and orthopaedic theatres. Group A received VR training in Year 1 and didactic training in Year 2, while Group B received the reverse. Radiation exposure was measured using Landauer Vision dosimeters. Participant feedback on satisfaction, engagement and confidence was collected through surveys. Data were analysed using paired *t*‐tests, generalised estimating equations and non‐parametric tests.

**Results:**

VR training significantly reduced radiation exposure compared to didactic training, with larger effect sizes per hour of training. Group A showed significant reductions during Year 1 when they received VR training (Year 2: didactic training), while Group B exhibited similar reductions during Year 2 when they underwent VR training (Year 1: didactic training). Group A, which received VR training in Year 1 followed by didactic training in Year 2, showed significant reductions in radiation exposure during Year 1. Group B, which received didactic training in Year 1 followed by VR training in Year 2, exhibited similar reductions during Year 2. Participant satisfaction and engagement were higher with VR training (*p* < 0.001), and confidence in applying safety practices increased significantly following VR training (*p* < 0.001). Group A reported these improvements after VR training in Year 1, while Group B experienced similar benefits after VR training in Year 2. Group A reported these improvements after VR training in Year 1, while Group B experienced similar benefits after VR training in Year 2.

**Conclusion:**

The RadSafe VR Program is more effective than traditional didactic training in reducing radiation exposure among medical professionals. VR training enhances radiation safety practices, improves participant satisfaction and increases confidence, offering a scalable and cost‐effective training solution.

## Graphical abstract

This study evaluates the comparative effectiveness of immersive virtual reality (VR) training and traditional didactic training on radiation safety among medical professionals, with a focus on reducing radiation exposure. A 2‐year randomised crossover trial involving 39 participants from cardiac catheterisation labs and orthopaedic theatres was conducted. Group A received VR training in year 1 and didactic training in year 2, while Group B received the reverse sequence. The results demonstrated significant reductions in radiation doses following VR training, particularly among interventional cardiologists and surgeons, with larger effect sizes compared to didactic methods. Additionally, the study found higher participant satisfaction, engagement and confidence with VR training, supporting its adoption as a more effective approach for improving radiation safety practices.

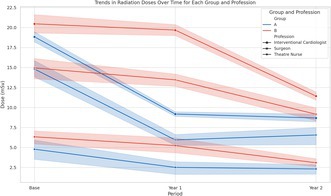

## 1 Introduction

Radiation safety is critical in medical settings where ionising radiation is routinely used, such as cardiac catheterization laboratories, orthopaedic surgery and operating theatres [1, 2, 3]. Chronic exposure to ionising radiation poses significant health risks to medical professionals, including increased risks of cancer, cataracts and other radiation‐induced conditions [3]. Effective radiation safety training is essential to equip healthcare workers with the knowledge and skills to implement safety protocols and minimise exposure [4, 5].

Traditional didactic training methods, often delivered through lectures and presentations, remain the standard approach for radiation safety education [6]. While effective in conveying theoretical knowledge, these methods frequently fall short in preparing medical professionals for practical application in clinical settings, where adherence to safety protocols is critical [7]. This limitation highlights the need for innovative training methods that enhance experiential learning and bridge the gap between theoretical understanding and practical implementation.

Virtual reality (VR) training offers a compelling solution by creating immersive and interactive environments that simulate real‐world conditions without exposing participants to actual risks [8]. Learners engage in hands‐on practice and realistic scenarios while receiving immediate feedback—elements proven to enhance knowledge retention and skill application. Nishi, Fujibuchi and Yoshinaga and Rainford et al. demonstrated that VR and augmented reality (AR) educational resources significantly improved understanding of scattered radiation behaviour [9, 10]. Radiography, radiology and medical students who used VR and AR developed deeper insights into radiation protection methods and reported increased confidence in applying these practices [10]. The ability to visualise radiation scatter interactively on web browsers without specialised devices further contributed to high motivation scores and overwhelmingly positive feedback [9].

Seymour et al. validated the effectiveness of VR in surgical training [11]. In their randomised, blinded study, residents trained with VR completed laparoscopic cholecystectomy 29% faster and made significantly fewer errors than their non‐VR‐trained counterparts. The VR‐trained group was six times less likely to make major errors during surgery, demonstrating that the skills acquired in a virtual environment translated effectively to clinical practice.

Koivisto et al. found that VR significantly improved clinical reasoning and decision‐making skills among healthcare students [12]. Similarly, Wojciechowski and Cellary reported that AR and VR environments increased learner engagement and knowledge retention, making them particularly effective for mastering complex topics such as radiation safety [13]. Rowe, Garcia and Rossi compared VR with physical simulation training for first‐year radiography students and found VR to be as effective as, or superior to, physical simulations in enhancing technical skills and understanding radiation safety protocols. VR also offered advantages in flexibility and scalability, which are critical for widespread implementation [14]. O'Connor et al. confirmed the utility of VR in their exploration of 3D VR simulations for radiation protection training, with students reporting higher confidence in applying their knowledge during clinical practice [10]. In a separate paper, O'Connor and Rainford identified performance improvements in 20 of 22 clinical assessment criteria for students trained using VR, with a control group comparison establishing a strong causal link between VR training and real‐world application [15].

John and Smyth demonstrated VR's utility in training radiology staff for mammographic screening, enabling the safe practice of sensitive procedures [16]. Harvard and Ryan observed improvements in both knowledge and practical application when VR simulations were integrated into undergraduate radiation protection education [17].

This growing body of evidence highlights VR's transformative potential in radiation safety education. By enabling learners to practice high‐risk scenarios in a controlled environment, VR leads to better retention of knowledge and more effective application of skills. Building on these findings, this study rigorously compares VR‐based radiation safety training with traditional didactic methods.

The primary objective of this study was to compare the effectiveness of VR‐based radiation safety training with traditional didactic methods in reducing radiation exposure to the lens of the eye among medical professionals. The secondary objective was to evaluate participant satisfaction, engagement and self‐reported confidence in applying radiation safety practices. This approach provides a comprehensive understanding of the efficacy and broader implications of VR training.

## 2 Methods

This 2‐year crossover trial compared the effectiveness of VR and traditional didactic training in radiation safety practices. A randomised design with a 1‐year washout period minimised variability, allowing participants to serve as their own control [17, 18, 19].

### 2.1 Recruitment

Participants were recruited through the hospital's education department. The study's voluntary nature, objectives and benefits were explained during an informational session, accompanied by detailed information sheets. Participants documented their previous radiation safety training, confirming no prior specialised education beyond standard professional training. This aligned with the European Commission's guidelines on Radiation Protection Education and Training (Radiation Protection No. 175) [20].

Participation in the study was entirely voluntary, with no impact on employment or evaluations. Participants reviewed consent forms, which emphasised their right to withdraw at any time.

The study recruited 39 medical professionals from a teaching hospital, split into two groups: Group A (3 Interventional Cardiologists, 6 Surgeons, 11 Theatre Nurses) and Group B (3 Interventional Cardiologists, 6 Surgeons, 10 Theatre Nurses). Inclusion criteria included no prior specialised training, roles involving radiation exposure and consent to wear dosimeters during both training sessions.

Interventional cardiologists worked in cardiac catheterization laboratories, while orthopaedic surgeons operated in orthopaedic theatres. Theatre nurses assisted in both settings. Participants were randomly assigned to groups using a computer‐generated sequence ensuring balanced professional representation. Case types involving radiation, such as hip, spinal and pelvic surgeries, or cardiac interventions, were documented.

### 2.2 Interventions

To compare the effectiveness of the two training methods, Group A first received VR‐based training during Year 1 and didactic training during Year 2. Conversely, Group B received didactic training in Year 1 and VR‐based training in Year 2. This crossover design ensured that each participant experienced both training methods. The VR training utilised the ‘RadSafe VR Program’, chosen for its immersive, interactive scenarios simulating real‐world conditions. This programme included specific scenarios for cardiac catheterization laboratories, hepatic angiography and cerebral angiography, allowing participants to engage in hands‐on practice and receive immediate feedback. This reinforcement of correct safety behaviours and techniques enhanced the understanding and application of radiation safety practices.

Additionally, the programme featured a comprehensive set of lessons providing detailed radiation data and visual reports on primary and scatter radiation behaviour based on machine positioning and settings. To prepare participants, the programme included a tutorial on hardware and software usage, enabling self‐directed training. The VR training duration was 2 h, divided into two 1‐h sessions over 2 days to optimise engagement and retention without overwhelming participants.

Guidance and explanations were provided by the Radiation Safety Officer (RSO) during VR training as needed. The RSO collected data, answered questions, and ensured participants were comfortable and confident using the software. The RSO also accessed learner feedback through its web portal for support.

The equipment for VR training included five Meta Quest 2 head‐mounted displays (HMDs) connected to VR‐ready laptops.

The didactic training consisted of classroom‐based lectures and presentations delivered by experienced RSOs. Content covered theoretical principles, practical safety measures and regulatory guidelines. The training duration was 4 h, divided into two 2‐h sessions over 2 days.

### 2.3 Dosimetry

Radiation exposure was measured using Landauer Vision dosimeters. Baseline readings were collected for 1 year prior to the study. Post‐training readings were recorded immediately after sessions and weekly throughout each study year. Participants were provided lead aprons, thyroid shields and glasses for the duration of the study. Dosimeter placement was standardised with permanent markings on the aprons to ensure consistent positioning.

To evaluate the efficiency of the training methods, the effect size per hour of training was calculated by dividing the effect size (Cohen's *d*) by the number of training hours for each method [21]. Generalised estimating equations (GEE) were used to account for the correlation of repeated measures and provide robust standard errors [22]. Effect sizes (Cohen's *d*) were calculated to quantify the magnitude of the differences between training methods [22]. Non‐parametric tests, such as the Wilcoxon signed‐rank test, were employed if data did not meet the assumptions for parametric tests [23]. Survey responses were analysed using descriptive statistics.

### 2.4 Surveys

Participant satisfaction, engagement and confidence in applying radiation safety practices were assessed using anonymous surveys conducted before and after each training session. Each participant was assigned an anonymized identification number to enable the tracking of individual responses while maintaining anonymity. These surveys utilised a standardised Likert scale ranging from 1 (low) to 5 (high) to measure overall satisfaction, perceived effectiveness, engagement and self‐reported confidence. By collecting responses in this manner, the study ensured unbiased feedback and provided a clear evaluation of how each training method—VR‐based or traditional didactic—impacted participants' experience and confidence in implementing radiation safety protocols.

To mitigate bias, randomisation was used to assign training orders, and a 1‐year washout period minimised carryover effects [18, 19]. Data analysts were blinded to group assignments, and consistent educational material was used for both VR and didactic sessions. Covariates were included in the statistical analysis to control confounding variables [18].

Ethics approval for this study was granted by the Japanese Institutional Review Board (approval no. JIRB‐2018‐4597) in 2018. Baseline radiation exposure data were collected from participants using Landauer Vision dosimeters for 1 year prior to the study's initiation, from January 2019 to December 2019. The 2‐year crossover trial began in January 2020, with Group A receiving VR training during the first year (January 2020–December 2020) followed by didactic training during the second year (January 2021–December 2021). Conversely, Group B received didactic training in Year 1 and VR training in Year 2. Post‐training dosimeter readings were recorded immediately after each training session and continued weekly throughout the study period. Final data analysis and reporting were conducted in 2022 to ensure a comprehensive evaluation of the training methods.

## 3 Results

To ensure a thorough analysis, case types were recorded throughout the study, and a chi‐squared statistical test was used to determine whether there were any significant differences in the distribution of case types between the different groups and across the 2 years of the study. The results indicated that the distribution of procedures was statistically similar between groups and years, with a *p*‐value of 0.049, indicating a marginally significant consistency across the study conditions.

### 3.1 Radiation Dose Reductions

Table 1 provides descriptive statistics of radiation doses (measured in mSv) for each profession, group and period (Base, Year 1 and Year 2), along with the effect sizes (Cohen's *d*) for changes in radiation doses over time. The mean radiation doses and effect sizes (Cohen's *d*) for each profession are summarised, highlighting significant dose reductions following VR training, particularly among interventional cardiologists and surgeons. For example, in Group A, the mean radiation dose for interventional cardiologists decreased significantly from baseline to Year 1 after VR training, with a large effect size (Cohen's *d* = 3.62). Surgeons in Group A experienced a reduction in radiation dose from 14.81 mSv at baseline to 5.94 mSv in Year 1, with a corresponding effect size of 2.58. Theatre nurses in Group A showed consistent reductions across all periods, with an effect size of 1.31 from baseline to Year 1. In Group B, similar trends were observed, with VR training in Year 2 (Year 1: didactic training) leading to more substantial reductions in radiation exposure compared to didactic training. The table underscores the substantial effectiveness of VR training in significantly lowering radiation exposure, with Group A showing reductions after VR training in Year 1 (Year 2: didactic training) and Group B showing reductions after VR training in Year 2 (Year 1: didactic training), as indicated by the larger effect sizes observed in the study.

**TABLE 1:** Radiation dose and effect sizes by profession, group and period.

Group	Profession	Period	Mean dose (mSv)	SD (mSv)	Effect size (Cohen's *d*)
A	Interventional cardiologist	Base	18.84	0.90	—
A	Interventional cardiologist	Year 1	9.18	0.41	3.62
A	Interventional cardiologist	Year 2	8.69	0.69	0.10
A	Surgeon	Base	14.81	1.28	—
A	Surgeon	Year 1	5.94	0.80	2.58
A	Surgeon	Year 2	6.56	1.17	−0.32
A	Theatre nurse	Base	4.78	1.74	—
A	Theatre nurse	Year 1	2.50	1.15	1.31
A	Theatre nurse	Year 2	2.30	0.88	0.13
B	Interventional cardiologist	Base	20.47	1.63	—
B	Interventional cardiologist	Year 1	19.69	0.97	0.22
B	Interventional cardiologist	Year 2	11.42	0.73	4.37
B	Surgeon	Base	14.95	1.58	—
B	Surgeon	Year 1	13.46	0.91	0.83
B	Surgeon	Year 2	9.16	1.02	3.35
B	Theatre nurse	Base	6.33	1.19	—
B	Theatre nurse	Year 1	5.23	1.43	0.58
B	Theatre nurse	Year 2	3.08	0.74	0.95

*Note:* Group A received VR training in Year 1 followed by didactic training in Year 2, while Group B received didactic training in Year 1 followed by VR training in Year 2.

Table 2 summarises the results of the GEE analysis, which accounts for the correlation of repeated measures to estimate the reduction in radiation doses across different professions and training groups. Negative coefficients indicate a reduction in radiation dose, with statistical significance indicated by *p*‐values. The analysis showed significant reductions in radiation doses across all professions after VR training, with *p*‐values ranging from 0.004 to < 0.001. For example, interventional cardiologists in Group A had a coefficient of −0.92 (*p* = 0.004), indicating a significant reduction in radiation dose following VR training. Similarly, surgeons and theatre nurses in both groups also experienced significant dose reductions, as reflected by the negative coefficients and corresponding *p*‐values in the GEE analysis.

**TABLE 2:** Generalised estimating equations analysis of radiation dose reductions. Group A received VR training in Year 1 followed by didactic training in Year 2, while Group B received didactic training in Year 1 followed by VR training in Year 2.

Profession	Group	Coefficient	SE	*z*	*p*
Interventional cardiologist	A	−0.92	0.32	−2.88	0.004
Interventional cardiologist	B	−0.86	0.30	−2.87	0.004
Surgeon	A	−1.24	0.35	−3.54	< 0.001
Surgeon	B	−1.19	0.34	−3.50	< 0.001
Theatre nurse	A	−0.64	0.28	−2.29	0.022
Theatre nurse	B	−0.56	0.26	−2.15	0.031

## 4 Non‐Parametric Tests

Table 3 presents the results of the Wilcoxon signed‐rank tests conducted for data that did not meet parametric assumptions to evaluate changes in radiation doses following the training interventions. The analysis confirmed significant reductions in radiation exposure after VR training, particularly for interventional cardiologists in Group A (*W* = 15, *p* = 0.023) and surgeons across both groups. The significance of dose reductions varied by profession and period, with VR training consistently proving more effective than didactic training in lowering radiation exposure. The table provides a comparison of radiation doses across different periods (Base, Year 1 and Year 2) for each profession and group, emphasising the effectiveness of VR training.

**TABLE 3:** Wilcoxon signed‐rank test results for radiation dose reduction. Group A received VR training in Year 1 followed by didactic training in Year 2, while Group B received didactic training in Year 1 followed by VR training in Year 2.

Profession	Group	Comparison	*W*	*p*
Interventional cardiologists	A	Base vs. Year 1 (VR)	15	0.023
	A	Year 1 vs. Year 2 (Didactic)	8	0.642
	B	Base vs. Year 1 (Didactic)	10	0.340
	B	Year 1 vs. Year 2 (VR)	17	0.013
Surgeons	A	Base vs. Year 1 (VR)	21	< 0.001
	A	Year 1 vs. Year 2 (Didactic)	9	0.154
	B	Base vs. Year 1 (Didactic)	14	0.056
	B	Year 1 vs. Year 2 (VR)	22	< 0.001
Theatre nurses	A	Base vs. Year 1 (VR)	33	0.001
	A	Year 1 vs. Year 2 (Didactic)	18	0.768
	B	Base vs. Year 1 (Didactic)	29	0.057
	B	Year 1 vs. Year 2 (VR)	34	0.014

### 4.1 Survey Findings

Table 4 summarises participant feedback on satisfaction, engagement and confidence levels associated with VR and didactic training methods, based on data collected from 39 participants. The results indicate significantly higher satisfaction and engagement with VR training than with didactic methods. Specifically, the average satisfaction rating for VR training was significantly higher (4.8 ± 0.4) than for didactic training (3.6 ± 0.7), and engagement levels were similarly higher during VR sessions, with an average rating of 4.7 ± 0.5 compared to 3.4 ± 0.8 for didactic training.

**TABLE 4:** Participant feedback on satisfaction, engagement and confidence.

Training method	Mean satisfaction rating	SD	*p*	Mean engagement rating	SD	*p*	Mean confidence rating (pre‐training)	Mean confidence rating (post‐training)	*p*
VR training	4.8	0.4	< 0.001	4.7	0.5	< 0.001	2.9	4.5	< 0.001
Didactic training	3.6	0.7	< 0.001	3.4	0.8	< 0.001	2.8	3.6	< 0.001

*Note:* This table includes participant feedback on satisfaction, engagement and confidence levels for each training method (VR and didactic). Group A received VR training in Year 1 and didactic training in Year 2. Group B received didactic training in Year 1 and VR training in Year 2.

Additionally, participants reported a significant increase in confidence in applying radiation safety practices following VR training, with the mean confidence rating increasing from 2.9 ± 0.6 before VR training to 4.5 ± 0.4 afterwards. In contrast, the didactic training showed a more modest improvement in confidence, from 2.8 to 3.6. Statistical analysis confirmed that all differences between VR and didactic training were significant (*p* < 0.001), demonstrating the superior effectiveness of VR training in enhancing participant experience and competence in radiation safety practices. These findings underscore the importance of immersive, interactive training environments in improving not only engagement and satisfaction but also practical confidence in safety‐critical skills.

**FIGURE 1:**
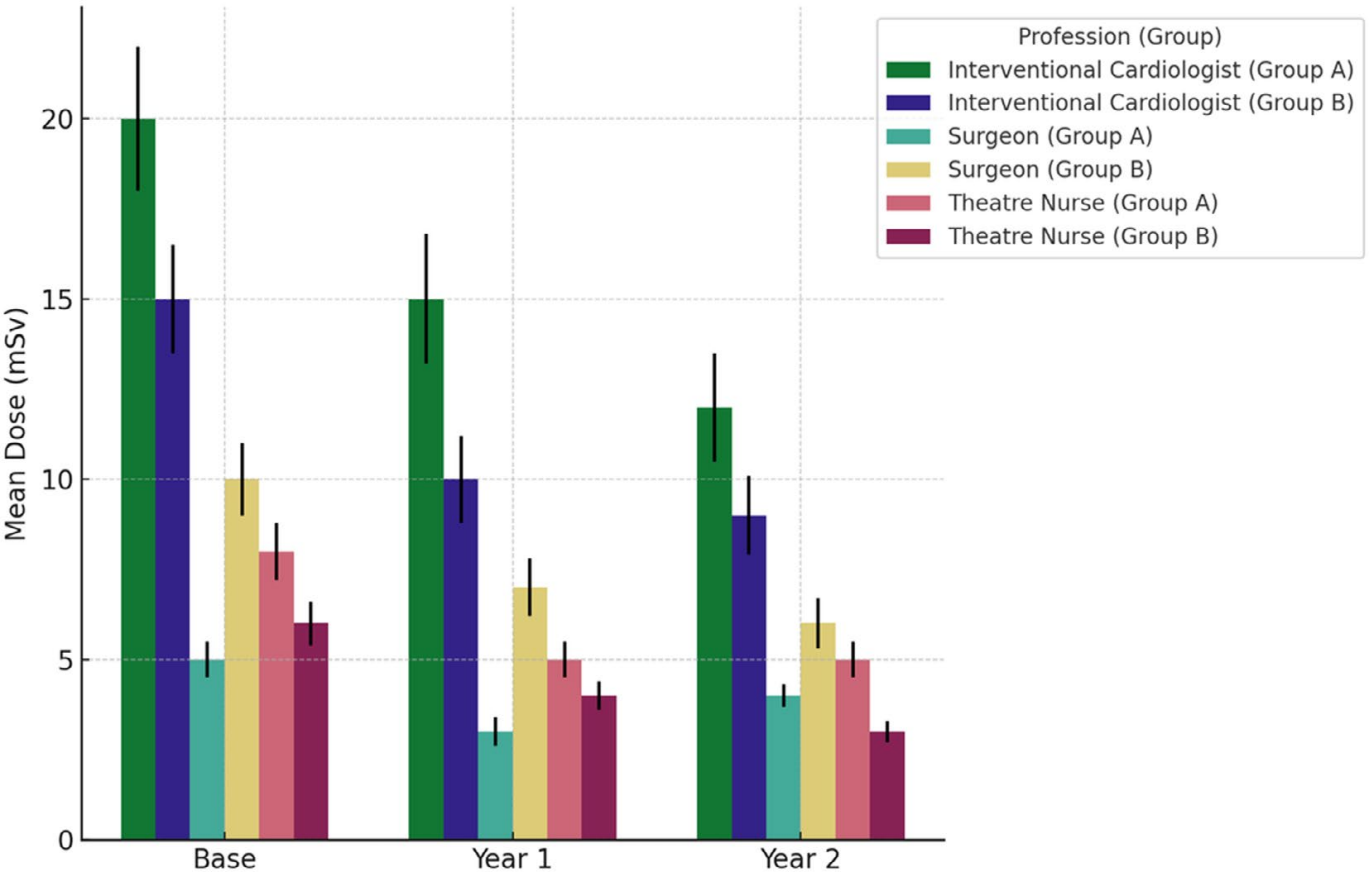
Radiation dose by profession, period and group.

**FIGURE 2:**
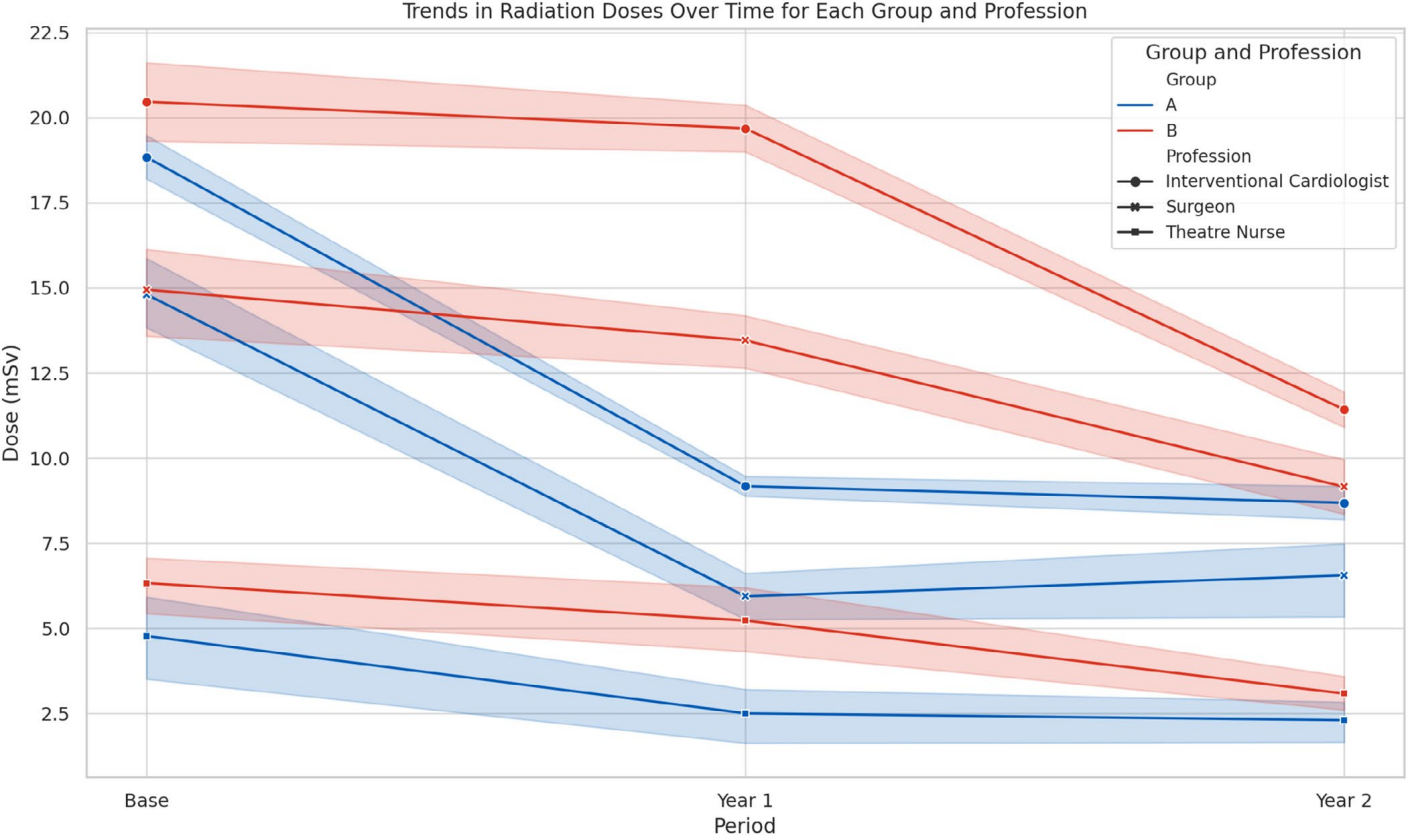
Trends in radiation dose reductions over time. Trends in radiation doses over time (Base, Year 1 and Year 2) are shown for each profession (interventional cardiologists, surgeons, theatre nurses) and training group (Group A and Group B). Lines represent the mean radiation doses, while shaded areas represent the standard deviation, indicating variability within the data.

Figure 1 illustrates the reductions in radiation doses over time (Base, Year 1 and Year 2) for each professional group (interventional cardiologists, surgeons and theatre nurses) within the two training groups (Group A and Group B). Group A received VR training in Year 1 followed by didactic training in Year 2, while Group B received didactic training in Year 1 followed by VR training in Year 2. The data are presented separately for each profession to highlight the differential impacts of VR and traditional didactic training methods on radiation exposure. Lines represent the mean radiation doses, and error bars indicate standard deviation, showing variability within the data. This breakdown ensures a clear understanding of how each profession benefited from the training interventions within their respective groups (Figure 2).

**FIGURE 3:**
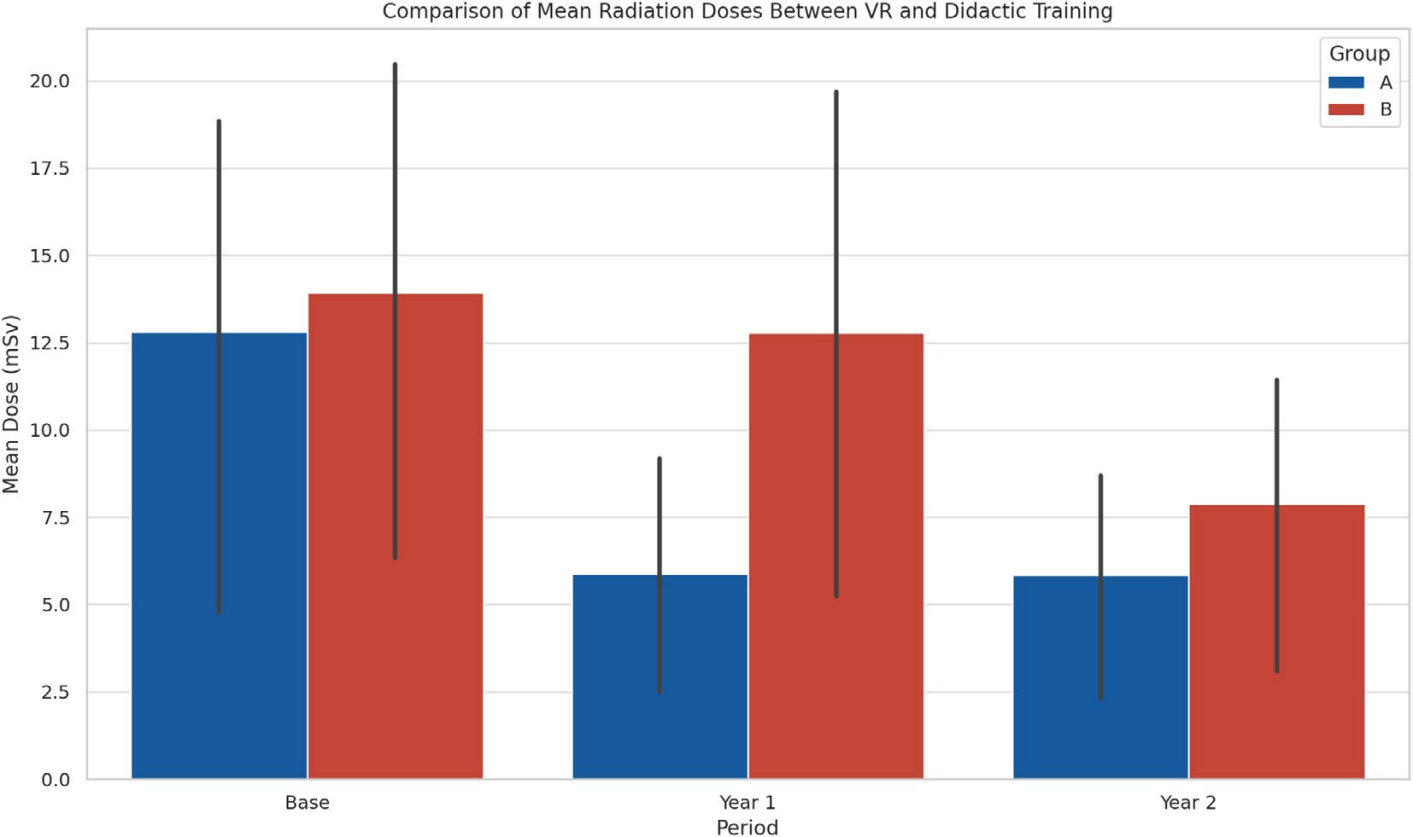
Comparison of mean radiation doses across training periods. This figure compares the mean radiation doses for each period (Base, Year 1 and Year 2) across training groups (Group A and Group B). Data are presented for each profession (interventional cardiologists, surgeons and theatre nurses) grouped together, enabling a direct comparison of the effectiveness of VR versus didactic training. The graph highlights the impact of each training method on radiation exposure over time.

Figure 3 compares the mean radiation doses for each period (Base, Year 1 and Year 2) across training groups (Group A and Group B). Group A received VR training in Year 1 followed by didactic training in Year 2, while Group B received didactic training in Year 1 followed by VR training in Year 2. Data are presented for each profession (interventional cardiologists, surgeons and theatre nurses) grouped together. Lines represent the mean radiation doses, and shaded areas indicate the standard deviation, highlighting the variation in radiation exposure within each profession over the study period. The graph enables a direct comparison of the effectiveness of VR versus didactic training, both within and across these professional groups, highlighting the impact of each training method on radiation exposure over time. Figures 1, 2, 3 demonstrate the effectiveness of VR training compared to traditional didactic methods in reducing radiation exposure among medical professionals. Participant feedback, detailed in Table 4, shows significantly higher satisfaction, engagement and confidence levels with VR training. The average satisfaction rating for VR was 4.8 (SD = 0.4) versus 3.6 (SD = 0.7) for didactic training (*p* < 0.001). Engagement ratings were similarly higher, with VR scoring 4.7 (SD = 0.5) compared to 3.4 (SD = 0.8) (*p* < 0.001). Confidence in applying radiation safety practices improved more substantially after VR training, increasing from 2.9 (SD = 0.6) to 4.5 (SD = 0.4) post‐training (*p* < 0.001), while didactic training showed a smaller improvement from 2.8 (SD = 0.7) to 3.6 (SD = 0.6). These statistically significant differences highlight the superior effectiveness of VR training in enhancing satisfaction, engagement and practical competence in radiation safety practices.

## 5 Discussion

This study employed a rigorous methodological approach to compare the effectiveness of VR‐based radiation safety training with traditional didactic training among interventional cardiologists, surgeons and theatre nurses. The primary objective was to evaluate the impact of these training methods on reducing radiation exposure, as measured by dosimeter readings. Secondary objectives included assessing participant satisfaction, engagement and self‐reported confidence in applying radiation safety practices.

Our findings indicate a significant reduction in radiation exposure following VR training, particularly in the first year for Group A and the second year for Group B. Participants reported significantly higher satisfaction and confidence levels after VR‐based training compared to traditional methods. The mean satisfaction rating for VR training was 4.8 (SD = 0.4) compared to 3.6 (SD = 0.7) for didactic training. Similarly, confidence in applying radiation safety practices increased more substantially with VR training, rising from 2.9 (SD = 0.6) to 4.5 (SD = 0.4), versus an increase from 2.8 (SD = 0.7) to 3.6 (SD = 0.6) with didactic training.

These findings align with existing literature, which highlights VR's advantages in enhancing user engagement and satisfaction. Nishi et al. demonstrated that VR and AR technologies significantly increased motivation and positive feedback among students learning radiological protection due to their interactive and immersive nature [9]. Similarly, Seymour et al. found that surgical residents who trained using VR reported higher satisfaction due to realistic simulations allowing hands‐on practice in a controlled environment [11].

The significant increase in confidence observed in this study underscores VR training's potential to enhance theoretical understanding and practical competence. This is particularly critical in high‐stakes environments like radiology, where confidence in safety protocols ensures better outcomes for patients and practitioners. Acosta et al. analysed the sustainability of VR training improvements. Their study demonstrates that integrating VR into radiography education leads to enhanced learning outcomes and skill retention, particularly in practical applications such as radiographic positioning and room set‐up [24]. They also highlight the hybrid training model's effectiveness in reducing costs and improving educational efficiency, suggesting that regular refresher courses and blended learning approaches could sustain these benefits over time.

Stratified analysis revealed that interventional cardiologists benefited the most from VR training, showing the largest reductions in radiation exposure, particularly in Group B, with an effect size of 4.37. Surgeons also saw significant benefits, with reductions in radiation exposure and effect sizes of 2.58 in Group A and 3.35 in Group B. However, while surgeons in Group A initially demonstrated significant dose reductions, an increase in Year 2, attributed to the transition from VR to less engaging didactic training, was noted. Interventional cardiologists and theatre nurses maintained more stable reductions, emphasising the importance of continuous immersive training like VR to sustain engagement and effectiveness.

These findings are supported by Graafland et al., who systematically reviewed serious games and VR in medical education, demonstrating their effectiveness in skill acquisition and retention in high‐stakes scenarios. Similarly, Hamilton et al. and Pottle emphasised VR's role in maintaining high engagement and consistent training outcomes [25, 26, 27].

Effect sizes calculated using Cohen's *d* confirmed VR's larger impact on radiation dose reductions compared to didactic training. For example, interventional cardiologists in Group A experienced a 51.3% reduction in doses from baseline to Year 1, decreasing from 18.84 to 9.18 mSv, with an effect size of 3.62. Surgeons showed reductions of 59.9% (14.81–5.94 mSv), and theatre nurses 47.7% (4.78–2.50 mSv). In Group B, interventional cardiologists saw reductions of 41.9% (19.69–11.42 mSv), while surgeons and theatre nurses showed 31.9% and 41.1% reductions, respectively. These trends align with international literature emphasising VR's efficacy in promoting adherence to safety protocols through immersive, hands‐on experiences [8, 26, 28, 29].

The cost‐efficiency of VR training is another notable advantage. Unlike traditional didactic training, which requires specialised facilities like catheterization laboratories, VR training can be conducted in standard rooms, eliminating the need for facility rentals. The self‐directed nature of VR also minimises instructor‐led sessions, reducing staff time and freeing resources for other tasks. Initial investments in VR hardware and software, such as the RadSafe VR Program, are modest and scalable, providing a cost‐effective long‐term solution for radiation safety training. Moreover, while VR training programmes require an upfront investment in technology, this cost is a small price to pay for a sustainable and statistically significant reduction in radiation exposure for nursing and medical professionals. The implications of limiting radiation exposure include lowering the risk of radiation‐induced malignancies and thereby adhering to the ‘as low as reasonably achievable’ (ALARA) principle. These benefits underscore the critical role of VR training in improving safety and minimising health risks associated with radiation exposure [26, 27, 28, 29, 30].

Participant feedback highlighted higher satisfaction and engagement with VR training compared to didactic methods. The interactive and immersive nature of VR enhances learner motivation, allowing participants to practice skills with immediate feedback, thereby increasing confidence and competence. These results align with studies showing VR's impact on improving training outcomes [11, 12, 27].

## 6 Limitations

This study has several limitations that should be acknowledged. First, the sample size was relatively small, comprising 39 participants from a single teaching hospital, which may limit the generalizability of the findings to broader, more diverse populations. Second, the study relied on a 2‐year crossover design with a 1‐year washout period, which, while minimising carryover effects, may have introduced temporal factors that influenced participant performance and engagement. Third, while the use of Landauer Vision dosimeters provided robust radiation exposure data, the accuracy of these measurements depends on consistent adherence to dosimeter placement protocols, which could not be independently verified outside supervised sessions. Additionally, the study did not account for potential variations in workload or case complexity between the two study years, which may have influenced radiation exposure levels. Finally, the reliance on self‐reported surveys for participant satisfaction and confidence introduces the potential for response bias, as participants may have over‐ or underestimated their experiences.

Future research addressing these limitations, including larger sample sizes, multicentre studies and more rigorous control of confounding variables, will be critical to validate and expand upon these findings.

## 7 Conclusion

This study demonstrates that VR training can significantly enhance satisfaction and confidence among medical professionals while contributing to reductions in radiation exposure. The tailored benefits observed across different professional groups highlight the versatility of VR training in addressing the specific needs of interventional cardiologists, surgeons, and theatre nurses. These findings underscore the potential of VR training programmes to improve radiation safety practices and preparedness in clinical settings, offering an effective approach to enhancing safety and competence in healthcare. The adoption of VR training also delivers cost savings by minimising resource use, reducing training time and eliminating the need for specialised facilities, making it a sustainable and efficient solution for healthcare education.

## Ethics Statement

The ethics approval (JIRB‐2018‐4597) was granted by the Osaka University Hospital Ethics Review Committee (Human Research).

## Conflicts of Interest

The authors declare no conflicts of interest.

## Data Availability

The data that support the findings of this study are available from the corresponding author, Wanjiku Mwangi, upon reasonable request. Due to privacy and ethical considerations, the data are not publicly available and will be provided only for legitimate research purposes.
